# Pandemic-related attitudes, stressors and work outcomes among medical assistants during the SARS-CoV-2 (“Coronavirus”) pandemic in Germany: A cross-sectional Study

**DOI:** 10.1371/journal.pone.0245473

**Published:** 2021-01-14

**Authors:** Annegret Dreher, Reinhard Pietrowsky, Adrian Loerbroks

**Affiliations:** 1 Institute of Occupational, Social and Environmental Medicine, Centre for Health and Society, Faculty of Medicine, University of Duesseldorf, Duesseldorf, Germany; 2 Institute of Experimental Psychology, Department of Clinical Psychology, University of Duesseldorf, Duesseldorf, Germany; Institute of Economic Growth, INDIA

## Abstract

**Background:**

The SARS-CoV-2 virus has spread rapidly around the globe since December 2019 creating much uncertainty among medical staff. Due to close patient contact, medical assistants are at increased risk of an infection. Several studies have investigated psychological consequences of the SARS-CoV-2 pandemic on medical staff, yet studies in the outpatient setting are scarce and studies addressing medical assistants are lacking. This study aimed to investigate pandemic-related stressors, attitudes, and work outcomes among medical assistants and to identify possible determinants.

**Methods:**

The population under study were medical assistants across entire Germany. A self-devised online questionnaire was published between April 7th, 2020, and April 14th. including questions on pandemic-related stressors, attitudes and work outcomes. Additionally, symptoms of depression and anxiety disorder were measured by PHQ-2 and GAD-2, respectively. Logistic regression was performed to identify possible determinants.

**Results:**

2150 medical assistants provided complete data (98.0% female, mean age 37.6 years). Major stressors were uncertainty about the temporal scope of the pandemic (95.1% agreement), about how to act correctly (77.5%), feelings of not being allowed to let patients down (75.9%), uncertainty about one’s financial situation (67.4%) and about contact persons for further information (67.1%). One third (29.9%) of the study population screened positively for depression and 42.6% for anxiety disorder. Feeling burdened by one’s financial situation was significantly associated with working in specialist practices (1.32 [1.08–1.62]), caring for children (1.51 [1.22–1.87]), depression (1.28 [1.01–1.62]), and anxiety disorder (1.93 [1.55–2.39]). Feeling burdened by thoughts about virus contraction at work was also significantly associated with working in specialist practices (1.33 [1.07–1.64]), caring for children (1.33 [1.07–1.66]), depression (1.54 [1.18–2.00]), and anxiety (4.71 [3.71–5.98]).

**Conclusions:**

This study provides novel evidence regarding major SARS-CoV-2 pandemic-related stressors among medical assistants and suggests need for special support for medical assistants caring for children and working in specialist practices.

## Introduction

Starting in Wuhan, Hubei province, China in December 2019, the severe acute respiratory syndrome coronavirus 2 (SARS-CoV-2) has quickly spread across the globe. On March 11th, the World Health Organization first spoke of the outbreak as a pandemic and added that it was the first pandemic ever to be caused by a coronavirus [1]. As of September 15th, 2020, over 29 million cases of SARS-CoV-2 infections and over 928,000 related deaths were confirmed worldwide with most cases and deaths being reported in Europe and the US. By that time, over 261,000 cases were confirmed in Germany [2].

In order to provide the best possible patient care during a pandemic, medical staff must be healthy, well-prepared for the situation and able to work in the best possible way. It is known from previous infectious disease outbreaks that these circumstances are not always met. In a study of 650 Chinese physicians and nurses working in intensive care units during the H1N1 (“swine flu”) pandemic in 2009/2010, Hu et al. reported that only slightly more than half had completed specific training before encountering H1N1 patients [3]. Only three quarters of physicians and nurses felt they had sufficient knowledge related to protecting themselves and others from the virus. The same study found a great lack of compliance regarding the use of personal protective equipment (PPE) among medical staff. This was mainly due to non-availability of PPE.

Several studies have investigated the well-being and worries of medical staff during various prior infectious disease outbreaks reporting high levels of anxiety [4], insomnia [5], and emotional stress as well as impaired quality of life [6]. In anticipation of a pandemic, medical staff–especially those working in hospitals–expect an increase in job strain and stress [7, 8]. Furthermore, research into prior infectious disease outbreaks suggests that medical staff is concerned about the job-related increased risk of infection and about passing on the infection to friends and family [7, 9]. It must be kept in mind that those previous studies mainly referred to the H1N1 (2009) and H5N1 (2003) outbreaks that had a significantly lower impact on Europe and the US with far fewer cases of human infections and deaths than the SARS-CoV-2 outbreak 2020 [10, 11].

Recently, first studies have investigated the impact of the current SARS-CoV-2 outbreak on the mental health of medical staff. Those studies reported high levels of psychological stress [12] as well as greater levels of fear, anxiety, and depression compared to the general population [13] and compared to administrative staff [14]. A German study by Kramer et al. investigated attitudes and stressors among hospital staff and identified nurses to report higher rates of stress compared to physicians. The same study found staff to more frequently feel stressed when working in departments that are particularly frequented by COVID-19 patients [15]. This study highlights that many different professional groups in the health care system are affected by the current pandemic, albeit to varying degrees.

In Germany, medical assistants (MAs) are the first professional group encountered by patients in primary care. In 2019, over 410,000 MAs worked mainly in outpatient and partially in inpatient settings. Medical assistants provide a large range of basic health care tasks with close patient contact, such as reception tasks, blood sampling, administration of injections, ECG recording, or blood pressure measurement [16, 17]. The risk of infection among this occupational group is exceptionally high due to their close patient contact and because primary care providers are usually the first contact for patients with symptoms similar to those of the coronavirus infection. However, to our knowledge no studies have yet shed light on well-being and worries of MAs during an infectious disease pandemic. Furthermore, research has not yet focused on the outpatient setting. This is, however, important as many patients report to general practices and other providers of outpatient care before they are possibly referred to hospitals. Hospitals may also treat the more severe cases of SARS-CoV-2 infections whereas in outpatient care milder symptoms are to be expected. This consequently leads to substantially different natures of stress in both settings. In hospitals, stress may stem from medical challenges whereas in outpatient settings stress may stem from lack of protective equipment and preparation for the pandemic situation

The aim of this study was therefore to, first, investigate the prevalence of attitudes, stressors, and work-related outcomes related to 2020 SARS-CoV-2 outbreak among MAs working mainly in outpatient but also in inpatient settings. The second aim was to identify potential determinants of those outcomes in order to identify potential high-risk groups that should be targeted through public health action.

## Materials and methods

### Study design and study sample

The study was approved by the ethics committee of the Medical Faculty of the University of Duesseldorf (study number 2020–899). Written consent was obtained by all study participants. A cross-sectional survey was conducted between April 7th and April 14th, 2020, using an online questionnaire distributed by the Association of Medical Professions (Verband medizinischer Fachberufe e.V.), Germany, on their webpage and social media. The Association of Medical Professions’ head office is located in Bochum, North Rhine-Westphalia, yet it acts nationwide across Germany. The distributed questionnaire therefore had the potential to reach out to members and followers of the association from all over Germany. A reminder was sent out on April 9th. All persons of legal age who were currently working as MAs were eligible for participation.

### Study instrument

The study instrument was developed by the authors in several steps. First, published literature was screened for validated questionnaires on pandemic-related stressors and attitudes among medical staff. Three questionnaires were found that seemed thematically suitable yet were not validated and two of them did not explicitly address medical staff. Items on perceived susceptibility to SARS-CoV-2 were adapted and modified from Liao et al. [18] and De Zwart et al. [19], items on preparedness and availability of PPE were adapted and modified from Hu et al. [3]. Further items e.g. on the perceived burdens due to childcare or the shortfall of colleagues were developed because of current media reports in Germany. The questionnaire was then discussed and optimized with experts of the Association of Medical Professions who contributed insights on additional current concerns of MAs. Experts of the association have not only worked as MAs themselves for many years but are in regular contact and exchange with MAs all over Germany through educational events, phone calls and own previous surveys among members.

The study questionnaire was set up using the UNIPARK software (Questback GmbH). The final instrument comprised questions on socio-demographic data, own contraction of SARS-CoV-2, contraction of SARS-CoV-2 among family, friends, and colleagues, and 16 questions on attitudes, stressors, and work-related outcomes during SARS-CoV-2 pandemic (see Tables 1 and 2 for the scope of constructs and wording of items). These questions covered, among others, the feeling of being prepared for treating SARS-CoV-2 patients, self-rated risk of infection, availability and perceived protection from personal protective equipment (PPE), and the burden of being uncertain about adequate behavior, contact persons, financial aspects, and the temporal scope of the SARS-CoV-2 pandemic. Participants were asked whether they agreed or disagreed with given statements on a 4-point Likert scale. Short version of the Patient Health Questionnaire (PHQ-2) [20] and the Generalized Anxiety Disorder questionnaire (GAD-2) [21] were used to measure depression and anxiety disorder, respectively.

**Table 1:** Socio-demographic characteristics of n = 2,150 study participants.

Characteristics	n (%)
Sex	
Male	42 (2.0)
Female	2,106 (98.0)
Non-binary	2 (0.1)
Age, mean (standard deviation)	37.6 (10.4)
18–32	783 (36.4)
33–42	668 (31.1)
43 and older	699 (32.5)
Permanent Partner	
Yes	1,804 (83.9)
No	346 (16.1)
Children under care in same household	
Yes	805 (37.4)
No	1,345 (62.6)
Highest level of education	
Low^1^	141 (6.6)
Intermediate^2^	1,596 (74.2)
High^3^	405 (18.8)
Other	8 (0.4)
Place of work	
General practice	1,022 (47.5)
Specialist practice	846 (39.3)
Medical care center	131 (6.1)
Hospital/clinic	78 (3.6)
Other	73 (3.4)
Self-rated health	
Very good	451 (21.0)
Good	1,273 (59.2)
Moderate	396 (18.4)
Bad	27 (1.3)
Very bad	3 (0.1)
Suspected or confirmed SARS-CoV-2 cases among friends and family	
Yes	349 (16.2)
No	1,801 (83.8)
Suspected or confirmed SARS-CoV-2 cases among colleagues	
Yes	331 (15.4)
No	1,819 (84.6)
Own previous infection with SARS-CoV-2	
Yes	22 (1.0)
No	2,128 (99.0)

1: Low: secondary modern school qualification (‘Haupt-/Volksschulabschluss’); 2: Intermediate: secondary school level I certificate (‘Mittlere Reife’, ‘Realschulabschluss’ or ‘Fachschulreife’); 3: High: general qualification for university entrance (‘Abitur’) or entrance qualification limited to universities of applied sciences (‘Fachhochschulreife’)

**Table 2:** SARS-CoV-2 related attitudes, stressors and work outcomes among n = 2,150 medical assistants in absolute numbers and percentages.

	Dichotomized scale for regression analysis		Original 4-point Likert Scale			
	Agree n(%)	Disagree n(%)	Strongly agree n(%)	Agree n(%)	Disagree n(%)	Strongly disagree n(%)
**SARS-CoV-2 related attitudes**						
The risk of contracting SARS-CoV-2 is higher for me than for a person of same age and sex from the general population	1,770 (82.3)	380 (17.7)	872 (40.6)	898 (41.8)	287 (13.3)	93 (4.3)
I feel sufficiently informed about dealing with SARS-CoV-2 patients by my employer	1,428 (66.4)	722 (33.6)	421 (19.6)	1,007 (46.8)	527 (24.5)	195 (9.1)
I feel sufficiently prepared for dealing with SARS-CoV-2 patients by my employer	1,301 (60.5)	849 (39.5)	278 (12.9)	1,023 (47.6)	621 (28.9)	228 (10.6)
My workload has increased due to the SARS-CoV-2 pandemic	1,076 (50.0)	1,074 (50.0)	436 (20.3)	640 (29.8)	838 (39.0)	236 (11.0)
I can use materials for personal protection at my work so that I feel sufficiently protected from contracting SARS-CoV-2	702 (32.7)	1,448 (67.3)	145 (6.7)	557 (25.9)	781 (36.3)	667 (31.0)
**SARS-CoV-2 related stressors**						
I am burdened by uncertainty about the temporal scope of the crisis	2,044 (95.1)	106 (4.9)	1,285 (59.8)	759 (35.3)	85 (4.0)	21 (1.0)
I am burdened by uncertainty about how to act correctly during the crisis	1,667 (77.5)	483 (22.5)	585 (27.2)	1,082 (50.3)	395 (18.4)	88 (4.1)
I am burdened by a feeling of not being able to let patients down during the crisis	1,630 (75.8)	520 (24.2)	756 (35.2)	874 (40.7)	415 (19.3)	105 (4.9)
I am burdened by the care situation of my children *(only for n = 805 MAs with children in their household)*	591 (73.4)	214 (26.6)	367 (45.6)	224 (27.8)	139 (17.3)	75 (9.3)
I am burdened by uncertainty about my financial situation during the crisis	1,448 (67.3)	702 (32.7)	726 (33.8)	722 (33.6)	512 (23.8)	190 (8.8)
I am burdened by uncertainty about contact persons during the crisis	1,444 (67.2)	706 (32.8)	478 (22.2)	966 (44.9)	575 (26.7)	131 (6.1)
I am burdened with thoughts of a possible infection with SARS-CoV-2 during work hours	1,413 (65.7)	737 (34.3)	474 (22.0)	939 (43.7)	554 (25.8)	183 (8.5)
I am burdened by the crisis-related shortfall of colleagues/staff at work	1,153 (53.6)	997 (46.4)	374 (17.4)	779 (36.2)	698 (32.5)	299 (13.9)
**SARS-CoV-2 related work outcomes**						
My employer takes the SARS-CoV-2 pandemic seriously	1,658 (77.1)	492 (22.9)	794 (36.9)	864 (40.1)	383 (17.8)	109 (5.1)
Due to the SARS-CoV-2 pandemic the care for patients with other diseases has been suffering	1,460 (67.9)	690 (32.1)	546 (25.4)	914 (42.5)	560 (26.0)	130 (6.0)
At my work all necessary materials for personal protection from SARS-CoV-2 are sufficiently available for me	516 (24.0)	1,634 (76.0)	122 (5.7)	394 (18.3)	888 (41.3)	746 (34.7)

### Statistical analysis

For this study, displaying the distribution of participants attitudes, stressors, and work-related outcomes was of special interest (i.e. research aim one). Descriptive analysis was carried out for all variables displaying absolute numbers and percentages for categorical variables. After checking for normal distribution of data, mean and standard deviations were reported for numeric variables. In order to address the second research aim, that is, identification of determinants of attitudes and stressors, logistic regression analysis was performed to analyze possible associations between dependent and independent variables. The independent variables were sociodemographic and work-related characteristics, suspected or confirmed SARS-CoV-2 cases among friends, family and colleagues, own SARS-CoV-2 infection, depression, and anxiety disorder (see Table 1). An important assumption when conducting logistic regression is the absence of outliers in the data which was met by all independent variables but age. One outlier was found for a participant aged 72. The variable age was therefore categorized into three even groups according to the tertile distribution. This categorized age variable was then included as independent variable into the multivariable models. A further assumption for logistic regression is the absence of multicollinearity between data. All independent variables were checked for multicollinearity by calculating Pearson’s correlation coefficients. All coefficients were below 0.45. Dependent variables comprised the 16 variables capturing attitudes, stressors, and work outcomes related to the SARS-CoV-2 pandemic (see Table 2). Associations were displayed as odds ratios (OR) with 95% confidence intervals (CI). In order to perform logistic regression, the originally 4-point answer scale of attitudes, stressors and work-related items was dichotomized into “agree” (score 1) and “disagree” (score 0). Cut-of values of ≥3 were used when assigning participants’ GAD-2 and PHQ-2 sum scores into categories ‘generalized anxiety disorder’ and ‘major depression’, respectively.

A multivariable model was run for each of the 16 dependent variables. For pandemic-related attitudes and stressors, all collected covariables except sex were included in the multivariable model (see Table 1). Sex was excluded from the analyses due to too few non-female participants (n = 44, 2.1%). For work-related outcomes only age and place of work were included. In subsequent sensitivity analysis, depression and anxiety disorder were removed from all models to reduce the likelihood that associations are spurious, that is, due to negative affect. All statistical analyses were done using IBM SPSS Statistics 25. The level of significance was set to α = 0.05.

## Results

### Study population

In total, 2,164 MAs participated in the survey. Due to questionnaire distribution via webpage and online groups, no exact response rates could be calculated. Two participants were underage, and 12 participants had missing data, leaving 2,150 participants with complete data that were included in all further analysis. Participants were mainly female (98%) with an age average of 37.6 years. Over 86% worked in an outpatient setting. As much as 349 MAs (16.2%) reported suspected or confirmed SARS-CoV-2 cases among friends and family, 331 (15.4%) among colleagues. Only 22 (1.0%) had already been infected themselves. Characteristics of the study population are displayed in Table 1.

### Descriptive analysis

SARS-CoV-2 related attitudes, stressors, and work-related outcomes are shown in Table 2. Sixty point five per cent of the participants stated that they felt sufficiently prepared by their employer for dealing with SARS-CoV-2 patients. Regarding self-rated risk of infection, 82.4% agreed that their risk of contracting SARS-CoV-2 was higher compared to a person of the same age and sex from the general population. Only 24.0% reported that all necessary materials for protection from SARS-CoV-2 were available for them. Major stressors were uncertainty about the temporal scope of the pandemic (95.1% agreement), uncertainty about how to act correctly (77.5%), a feeling of not being allowed to let patients down (75.9%), uncertainty about one’s financial situation (67.4%), and uncertainty about contact persons (67.1%). According to PHQ-2, one third (29.9%) of the study population screened positively for depression and, according to GAD-2, 42.6% screened positively for anxiety disorder.

### Logistic regression results

#### SARS-CoV-2 related attitudes

Older participants were more likely to perceive a higher personal risk of SARS-CoV-2 contraction (OR 1.38 [CI 1.04–1.83]). Those caring for children and those with higher education were less likely to feel sufficiently protected from SARS-CoV-2, whereas participants with good self-rated health were more likely to feel sufficiently protected. Medical assistants working in specialist practices, medical care centers, or in hospitals were less likely to report an increased workload compared to MAs working in general practices. On the other hand, workload increased for MAs who reported suspected or confirmed SARS-CoV-2 cases among their colleagues (1.64 [1.26–2.14]). Medical assistants working in specialist practices felt less prepared (0.65 [0.53–0.79]) and less informed (0.54 [0.44–0.66]) by their employer about how to deal with SARS-CoV-2 patients than MAs working in general practices. The same pattern was observed for MAs with depression or anxiety disorder. (Table 3)

**Table 3:** Logistic regression results for SARS-CoV-2 related attitudes among medical assistants (n = 2,150).

	SARS-CoV-2 related attitudes									
	Higher perceived risk of contraction		Feeling of sufficient protection from infection		Feeling sufficiently prepared		Feeling sufficiently informed		Increased workload due to pandemic	
	OR	95% CI	OR	95% CI	OR	95% CI	OR	95%CI	OR	95%CI
Age										
33–42 (vs. 18–32)	1.15	0.86–1.54	1.01	0.79–1.29	0.97	0.77–1.23	0.96	0.75–1.22	1.16	0.92–1.48
43 and older (vs. 18–32)	1.38	1.04–1.82	0.89	0.71–1.13	0.94	0.75–1.17	0.87	0.69–1.10	1.02	0.81–1.27
Permanent Partner										
Yes (vs. no)	0.91	0.66–1.24	0.90	0.70–1.16	0.93	0.73–1.20	0.89	0.69–1.16	0.69	0.54–0.89
Children under care in same household										
Yes (vs. no)	1.00	0.77–1.29	0.76	0.61–0.93	0.96	0.79–1.18	1.03	0.84–1.28	1.03	0.84–1.26
Highest level of education										
Intermediate^2^ (vs. low^1^)	0.90	0.56–1.43	0.68	0.48–0.97	0.81	0.56–1.16	0.90	0.62–1.31	0.89	0.62–1.27
High^3^ (vs. low^1^)	0.92	0.55–1.55	0.62	0.41–0.93	0.70	0.47–1.05	0.74	0.49–1.12	0.72	0.48–1.08
Place of work										
Specialist practice (vs. general practice)	0.75	0.59–0.95	0.86	0.70–1.05	0.65	0.53–0.79	0.54	0.44–0.66	0.37	0.25–0.55
Medical care center (vs. general practice)	1.27	0.74–2.15	0.93	0.62–1.39	0.87	0.59–1.28	0.45	0.31–0.66	0.47	0.29–0.77
Hospital/clinic (vs. general practice)	1.26	0.64–2.50	1.27	0.77–2.08	0.66	0.40–1.07	0.92	0.54–1.57	0.56	0.34–0.91
Other (vs. general practice)	1.31	0.64–2.71	0.94	0.55–1.58	0.64	0.39–1.05	0.55	0.34–0.91	0.56	0.34–0.91
Self-rated health										
Good (vs. bad)	0.71	0.52–0.99	1.54	1.19–2.00	1.44	1.15–1.81	1.44	1.15–1.82	0.92	0.73–1.17
SARS-CoV-2 cases among friends and family										
Yes (vs. no)	1.31	0.94–1.82	1.03	0.80–1.34	0.92	0.72–1.18	0.81	0.63–1.04	1.11	0.86–1.42
SARS-CoV-2 cases among colleagues										
Yes (vs. no)	1.11	0.79–1.57	1.05	0.80–1.37	0.70	0.55–0.91	0.82	0.63–1.07	1.64	1.26–2.13
Own previous infection with SARS-CoV-2										
Yes (vs. no)	0.80	0.26–2.45	0.59	0.21–1.66	0.86	0.35–2.09	0.94	0.38–2.36	3.41	1.10–10.60
Depression										
Yes (vs. no)	1.29	0.96–1.73	0.69	0.55–0.88	0.66	0.53–0.82	0.60	0.48–0.75	1.15	0.92–1.43
Anxiety Disorder										
Yes (vs. no)	1.52	1.17–1.98	0.58	0.47–0.71	0.56	0.46–0.69	0.66	0.54–0.81	2.10	1.71–2.57

OR Odds ratio; CI Confidence interval; 1: Low: secondary modern school qualification (‘Haupt-/Volksschulabschluss’); 2: Intermediate: secondary school level I certificate (‘Mittlere Reife’, ‘Realschulabschluss’ or ‘Fachschulreife’); 3: High: general qualification for university entrance (‘Abitur’) or entrance qualification limited to universities of applied sciences (‘Fachhochschulreife’)

#### SARS-CoV-2 related stressors

MAs in the youngest age group were more likely to be burdened by thoughts about contraction at their workplace. The same was observed for participants caring for children, those working in specialist practices, those with depression, and those with anxiety disorder. The pandemic-related shortfall of colleagues was more stressful to MAs working in medical care centers (1.50 [1.01–2.22]) and hospitals (1.73 [1.03–2.93]) compared to general practices. Feeling burdened by the childcare situation was less common among MAs 43 years and older compared to younger MAs (0.23 [0.14–0.39]). Uncertainty about acting correctly was significantly less likely among MAs 43 and older, those with intermediate and high education, and those with good health. In contrast, MAs caring for children, those with depression, and those with anxiety disorder were more likely to report uncertainties about acting correctly. Uncertainty about one’s financial situation was more likely among MAs either caring for children, working in specialist practices, suffering from depression, or suffering from anxiety disorder. (Table 4)

**Table 4:** Logistic regression results for SARS-CoV-2 related stressors among medical assistants (n = 2,150).

	SARS-CoV-2 related stressors													
	Thoughts about contraction at workplace			Shortfall of colleagues			Childcare situation*		Not being able to let patients down			Uncertainty about acting correctly		
	OR	95% CI		OR	95% CI		OR	95% CI	OR		95%CI	OR		95%CI
Age														
33–42 (vs. 18–32)	0.72	0.56–0.94		1.01	0.79–1.28		1.08	0.65–1.82	0.73		0.56–0.96	0.80		0.60–1.07
43 and older (vs. 18–32)	0.91	0.72–1.16		1.05	0.84–1.31		0.23	0.14–0.39	0.87		0.67–1.12	0.67		0.52–0.87
Permanent Partner														
Yes (vs. no)	1.11	0.85–1.46		1.09	0.85–1.40		1.77	1.00–3.13	0.87		0.65–1.16	1.09		0.82–1.45
Children under care in same household														
Yes (vs. no)	1.33	1.07–1.66		0.92	0.75–1.13		-	-	1.17		0.93–1.48	1.37		1.07–1.74
Highest level of education														
Intermediate^2^ (vs. low^1^)	0.75	0.50–1.13		0.87	0.61–1.24		0.91	0.45–1.86	0.70		0.45–1.11	0.47		0.28–0.80
High^3^ (vs. low^1^)	0.66	0.42–1.03		0.93	0.62–1.39		1.11	0.50–2.45	0.52		0.32–0.85	0.37		0.21–0.65
Place of work														
Specialist practice (vs. general practice)	1.33	1.07–1.64		1.16	0.96–1.41		1.38	0.95–1.99	0.76		0.60–0.95	1.04		0.83–1.31
Medical care center (vs. general practice)	0.98	0.65–1.50		1.50	1.01–2.22		0.75	0.34–1.61	0.93		0.59–1.46	0.89		0.57–1.41
Hospital/clinic (vs. general practice)	1.40	0.82–2.39		1.73	1.03–2.93		1.49	0.49–4.53	0.68		0.39–1.17	1.05		0.59–1.89
Other (vs. general practice)	1.42	0.81–2.51		1.19	0.72–1.98		1.72	0.63–4.67	0.81		0.45–1.44	0.75		0.43–1.33
Self-rated health														
Good (vs. bad)	0.55	0.41–0.73		0.80	0.63–1.02		0.91	0.57–1.45	0.84		0.63–1.14	0.62		0.45–0.86
SARS-CoV-2 cases among friends and family														
Yes (vs. no)	1.21	0.92–1.60		0.83	0.64–1.06		0.79	0.50–1.24	1.11		0.83–1.50	1.08		0.80–1.47
SARS-CoV-2 cases among colleagues														
Yes (vs. no)	1.23	0.92–1.64		2.64	2.00–3.49		1.66	0.93–2.98	1.28		0.93–1.76	1.25		0.90–1.72
Own previous infection with SARS-CoV-2														
Yes (vs. no)	1.05	0.38–2.88		1.75	0.66–4.62		0.18	0.04–0.87	0.95		0.33–2.74	1.32		0.42–4.16
Depression														
Yes (vs. no)	1.54	1.18–2.00		1.62	1.29–2.02		1.14	0.72–1.80	1.80		1.34–2.41	1.66		1.23–2.25
Anxiety Disorder														
Yes (vs. no)	4.71	3.71–5.98		2.27	1.85–2.78		1.83	1.23–2.71	2.96		2.29–3.82	2.63		2.02–3.42
	SARS-CoV-2 related stressors													
	Uncertainty about contact persons					Uncertainty about financial situation					Uncertainty about temporal scope			
	OR		95% CI			OR				95% CI	OR		95% CI	
Age														
33–42 (vs. 18–32)	1.14		0.88–1.46			1.05				0.82–1.34	0.65		0.38–1.13	
43 and older (vs. 18–32)	1.01		0.80–1.27			0.90				0.72–1.13	0.55		0.34–0.89	
Permanent Partner														
Yes (vs. no)	1.02		0.79–1.33			0.94				0.73–1.21	1.21		0.73–2.00	
Children under care in same household														
Yes (vs. no)	1.16		0.94–1.44			1.51				1.22–1.87	2.45		1.48–4.06	
Highest level of education														
Intermediate^2^ (vs. low^1^)	0.78		0.53–1.16			0.77				0.52–1.13	1.42		0.71–2.86	
High^3^ (vs. low^1^)	0.60		0.39–0.92			0.75				0.49–1.16	1.64		0.72–3.73	
Place of work														
Specialist practice (vs. general practice)	1.06		0.86–1.30			1.32				1.08–1.62	0.80		0.52–1.23	
Medical care center (vs. general practice)	0.70		0.47–1.04			0.96				0.65–1.43	0.83		0.34–1.92	
Hospital/clinic (vs. general practice)	1.12		0.67–1.89			0.79				0.49–1.30	1.43		0.41–4.93	
Other (vs. general practice)	0.85		0.50–1.44			1.112				0.66–1.88	0.78		0.27–2.27	
Self-rated health														
Good (vs. bad)	0.66		0.50–0.86			0.75				0.58–0.97	0.77		0.41–1.42	
SARS-CoV-2 cases among friends and family														
Yes (vs. no)	1.04		0.80–1.35			0.98				0.75–1.26	0.85		0.49–1.45	
SARS-CoV-2 cases among colleagues														
Yes (vs. no)	1.19		0.90–1.58			0.95				0.73–1.24	0.81		0.46–1.41	
Own previous infection with SARS-CoV-2														
Yes (vs. no)	2.15		0.69–6.67			0.87				0.35–2.17	0.58		0.13–2.71	
Depression														
Yes (vs. no)	1.76		1.37–2.26			1.28				1.01–1.62	1.99		1.04–3.79	
Anxiety Disorder														
Yes (vs. no)	2.38		1.91–2.97			1.93				1.55–2.39	2.70		1.56–4.70	

OR Odds ratio; CI Confidence interval; 1: Low: secondary modern school qualification (‘Haupt-/Volksschulabschluss’); 2: Intermediate: secondary school level I certificate (‘Mittlere Reife’, ‘Realschulabschluss’ or ‘Fachschulreife’); 3: High: general qualification for university entrance (‘Abitur’) or entrance qualification limited to universities of applied sciences (‘Fachhochschulreife’)

*only for n = 805 MAs with children under care in their household

#### SARS-CoV-2 related work outcomes

Regarding pandemic related work outcomes, significant differences between workplaces were found. Compared to general practices, MAs working in specialist practices were less likely to report that their employer took the pandemic seriously (0.56 [0.45–0.70]) but were also less likely to report that patient care suffered due to the pandemic (0.60 [0.49–0.73]). Medical assistants working in hospitals were more likely to confirm that all necessary materials for personal protection from SARS-CoV-2 were sufficiently available for them (1.91 [1.18–3.09]).

All abovementioned patterns of associations were largely supported by sensitivity analysis that excluded depression and anxiety disorder from the multivariable models (see supplemental material). Only for self-rated health, effect estimates decreased by about 0.2 for most stressors. Estimates increased by about 0.4 for the feeling of being sufficiently protected, prepared, and informed.

## Discussion

This study is the first to describe attitudes, stressors, and work outcomes of medical assistants in Germany due to and during the 2020 SARS-CoV-2 outbreak. The first aim of the study was to describe prevalences of attitudes, stressors, and work outcomes. All investigated stressors were reported frequently. The stressors most MAs agreed to were uncertainty about the pandemic’s temporal scope, uncertainty about how to act correctly during the crisis and a feeling of not being able to let patients down. Regarding SARS-CoV-2 related attitudes, 82.3% of participants stated that they perceive a higher risk of infection compared to the general population which is in line with the original hypothesis that MAs are at exceptionally high risk of infection. Other studies have also reported the own risk of infection with SARS-CoV-2 to be a major stressor among medical staff [22, 23]. Almost 40% of MAs did not feel sufficiently prepared by their employer for dealing with SARS-CoV-2 patients and 33.6% did not feel sufficiently informed. Regarding SARS-CoV-2 related work outcomes, less than a quarter of MAs stated that enough PPE were available for them to use and over two-third of MAs felt that patient care was suffering for patients with other diseases than COVID-19.

The levels of uncertainty and agreement to different stressors in this study were very high. The study sample in this study, medical assistants, were mainly women working in an outpatient setting and performing non-physician tasks. Several studies among medical staff during the SARS-CoV-2 pandemic have found women to suffer from higher levels of distress than men [5, 24]. Other studies reported nursing staff to feel more anxious during the pandemic than physician staff [22]. With respect to the outpatient setting, only one Chinese study has explicitly investigated outpatient healthcare staff during the SARS-CoV-2 pandemic, yet focusing on organizational changes rather than the psychological effects [25]. Possibly the high agreement to stressors in this study may be due to the fact that the majority of participants worked in an outpatient setting and thereby faced poorer preparation for the SARS-CoV-2 pandemic (e.g. lack of information and PPE) than staff working in the hospital setting. Hospitals may have greater PPE supplies and units specifically trained for the treatment of infectious disease patients. This hypothesis is supported by the finding in this study that participants from hospital were less likely to mention a lack of PPE–yet the absolute number of participants from hospitals was too low in this study to draw further conclusions.

The study findings underline a need for employer support in crisis management and crisis communication. It remains unclear whether employers themselves feel sufficiently informed and prepared by e.g. the Public Health Department or the Association of Statutory Health Insurance Physicians and are therefore able to pass on knowledge to their employees.

All surveyed SARS-CoV-2-related stressors were reported very frequently, especially uncertainty about how to act correctly and uncertainty about contact persons for further information on how to act. Only those MAs who know how to act correctly can reliably protect themselves and others from infection, therefore clear guidelines for action are required, which must be bindingly communicated by superior health institutions and must be known to all MAs. Reported uncertainties, feelings of being at higher risk of infection, and worries about infection at one’s workplace may be additionally fostered by the non-availability of PPE, as only 24% of MAs stated that PPE were available in sufficient quantity for them to use at their workplace. A study among Chinese healthcare workers during the early SARS-CoV-2 pandemic found a shortage of PPE to be independently associated with anxiety levels [26].

About one third of MAs screened positive for depression and an even higher number for generalized anxiety disorder. These numbers are much higher than those reported in a study by Tan et al. among Chinese frontline workers or by Zhang et al. among the Chinese general population during the SARS-CoV-2 outbreak [13, 27] Tan et al. used different tools to measure depression and anxiety and argued that frontline staff might receive more formal psychological support and therefore show lower rates of depression and anxiety compared to non-frontline staff. The results of the present study might also be explained by this hypothesis, as many MAs stated uncertainty about how to act correctly and felt insufficiently prepared indicating that they had not received any form of support.

The second aim of the study was to identify possible determinants of attitudes, stressors and work outcomes related to the SARS-CoV-2 outbreak among medical assistants. Logistic regression analysis revealed significant associations between personal characteristics, health-related characteristics and reported attitudes and stressors. Medical assistants working in specialist practices, those caring for children and those suffering from either depression or anxiety disorder were more likely to report various stressors than their counterparts. Regarding specific outcomes, MAs working in specialist practices were less likely to report an increase of workload due to the SARS-CoV-2 pandemic compared to those working in general practices. A possible explanation may be that general practices in Germany are usually contacted first when patients feel sick and are commonly visited for symptoms such as fever and cough, which are key symptoms of SARS-CoV-2, infections. These findings are in line with the observation that MAs from specialist practices feel less informed and prepared by their employer about SARS-CoV-2 compared to those in general practices. Employers in specialist practices might not feel the necessity of preparation because they are not seen as the first contact points for consultations.

Medical assistants with children under care in their household felt significantly less protected by PPE, significantly more burdened by thoughts about possible contraction at work and about their financial situation. They also felt uncertainty about correct behavior to a greater extent. These findings are in line with a Taiwanese study who found medical staff to suffer from higher stress levels during the SARS-CoV-2 pandemic when caring for underage children [23]. A study by Cai et al. found that the safety of participants’ families was inversely associated with stress among hospital staff during the SARS-CoV-2 pandemic [22]. Likewise, another Taiwanese study found the fear of transmitting SARS-CoV-2 to relatives to be one of the greatest stressors among hospital staff [23]. People caring for children are expected to have a particularly high level of responsibility for themselves and their families and may therefore be more concerned about own contraction and contraction of family members. In this study, older MAs felt less burdened by their childcare situation. Possibly, their children are older and more independent compared to those of younger MAs.

In this study, participants with better self-rated health almost consistently showed better outcomes. These participants might feel more confident about their health and might less likely fear an own contraction of SARS-CoV-2. A systematic review by Luo et al. also found poorer health to be a risk factor when investigating the psychological impact of the SARS-CoV-2 pandemic on medical staff and the general population [28]. In contrast, participants with depression or anxiety disorder almost consistently showed higher degrees of uncertainty, felt less informed, less prepared, and less protected by PPE, possibly because these participants feel fundamentally more insecure and tend to rate the SARS-CoV-2 outbreak and its consequences more negatively.

### Strengths and limitations

A major strength of this study is that it was conducted during peak times of the SARS-CoV-2 outbreak in Germany and therefore probably captured stressors and attitudes with very little potential for recall bias. A high participation rate was achieved, and, to the authors knowledge, this is the first study examining stressors of MAs during an infectious outbreak although this population is at exceptionally high risk of infection.

Yet, several limitations of the study must be discussed. A major limitation is the study’s cross-sectional design which does not allow to interpret associations as causal. The exact number of MAs who received the invitation link to participate in the survey could not be tracked and therefore no response rate could be calculated. It must also be considered that despite the reach of the Association of Medical Professions, probably only a fraction of MAs in Germany has received the study link as not all of them are either members of the Association or following its’ activities. As regards representativeness, the study sample was comparable to a previous representative study among MAs in Germany in respect of sex distribution and mean participant age [29]. Yet, compared to the official figures of the German Employment Agency, MAs within the age group of 25–54 took part in this study somewhat more frequently than participants 55 and older [16]. This may be due to the online distribution of the study’s invitation link as online content and social media usage is more common among younger people. A further possible limitation is that over 99% of the study sample had not contracted SARS-CoV-2 prior to or during the point of investigation. It remains unclear whether this is due to low infection rates across the German population in early April 2020 or if those affected by SARS-CoV-2 were systematically missed during participant recruitment.

Finally, a self-developed questionnaire was used that covered many different stressors and attitudes that had partially been reported in previous studies and other stressors that had not been investigated so far. No validated questionnaires were available at the point of survey and, due to time constraints, it was not possible to explore our instrument’s psychometric properties. No pilot testing of the instrument was done before distribution, but questionnaire adaption by experts from the field.

## Conclusion

In conclusion, the results of this study show a low level of preparedness and high levels of uncertainty among MAs in Germany regarding the SARS-CoV-2 pandemic. Medical assistants working in specialist practices, those caring for children and those suffering from either depression or anxiety disorder were particularly likely to feel uncertain about the situation. Preparation strategies for future infectious disease outbreaks could be, firstly, keeping enough personal protective equipment in stock, secondly, to strengthen disease control measures within the medical assistant training program and lastly to assure clear and swift communication by politicians and associations about all necessary information in case of an infectious disease outbreak. Counseling services should be offered to all MAs in exceptional situations for treatment of distress. Financial worries could be buffered by immediate offers of financial support for short-time workers. Furthermore, all necessary measures should be taken by the government to prevent further closures of daycare facilities so that childcare is always secure.

## Supporting information

S1 File (PDF)

S2 File (PDF)

S1 Table Sensitivity analysis: Logistic regression results for SARS-CoV-2 related attitudes among medical assistants (n = 2,150) without adjusting for depression and anxiety disorder. (DOCX)

S2 Table Sensitivity analysis: Logistic regression results for SARS-CoV-2 related stressors among medical assistants (n = 2,150) without adjusting for depression and anxiety disorder. (DOCX)
