# Supplementary material for: Pandemic-related attitudes, stressors and work outcomes among medical assistants during the SARS-CoV-2 (“Coronavirus”) pandemic in Germany: A cross-sectional Study
Source: PLoS One. 2021 Jan 14;16(1):e0245473. doi: 10.1371/journal.pone.0245473 (PMC7808691; doi:10.1371/journal.pone.0245473)
Supplement: S1 File — (PDF) [file pone.0245473.s001.pdf]

# Questionnaire on the SARS-CoV-2 ("Coronavirus") pandemic

1. **Sex**
   - Male
   - Female
   - Non-binary

2. **Year of birth**
   - __________

3. **Do you have a permanent partner?**
   - Yes
   - No

4. **Do you have persons in your household who need care?**
   - Yes, children under care
   - Yes, adults under care
   - Yes, both, children and adults under care
   - No

5. **What is your highest level of education?**
   - Secondary modern school qualification
   - Secondary school level I certificate
   - General qualification for university entrance or entrance qualification limited to universities of applied sciences
   - Other (e.g. obtained abroad)

6. **Are you currently working as a (dental) medical assistant?**
   - Yes, as a medical assistant
   - Yes, as a dental assistant
   - No, working as other: ______________________
   - I am currently not employed (e.g. looking for work, maternity leave)

7. **What is your current place of work?**
   - Medical care center
   - Hospital/clinic
   - Rehabilitation area
   - General practice
   - Dental practice
   - Specialist practice with the following specialization: ____________________________
   - Other: ________________________

8. **In general, how would you describe your state of health?**
   - Very good
   - Good
   - Moderate
   - Bad
   - Very bad

9. **There are suspected or confirmed SARS-CoV-2 cases among my friends and family.**
   - Yes
   - No

10. **There are suspected or confirmed SARS-CoV-2 cases among my colleagues.**
    - Yes
    - No

11. **You yourself have already tested positive for SARS-CoV-2.**
    - Yes
    - No

**The following questions refer to the current SARS-CoV-2 pandemic ("coronavirus"). Please indicate to what extent you agree with the following statements.**

| | **Strongly Disagree** | **Disagree** | **Agree** | **Strongly agree** |
|---|---|---|---|---|
| I feel sufficiently informed about dealing with SARS-CoV-2 patients by my employer | | | | |
| I feel sufficiently prepared for dealing with SARS-CoV-2 patients by my employer | | | | |
| My employer takes the SARS-CoV-2 pandemic seriously | | | | |
| The risk of contracting SARS-CoV-2 is higher for me than for a person of same age and sex from the general population | | | | |
| My workload has increased due to the SARS-CoV-2 pandemic | | | | |
| Due to the SARS-CoV-2 pandemic the care for patients with other diseases has been suffering | | | | |
| At my work all necessary materials for personal protection from SARS-CoV-2 are sufficiently available for me | | | | |
| I can use materials for personal protection at my work so that I feel sufficiently protected from contracting SARS-CoV-2 | | | | |
| I am burdened with thoughts of a possible infection with SARS-CoV-2 during work hours | | | | |
| I am burdened by the crisis-related shortfall of colleagues/staff at work | | | | |
| I am burdened by the care situation of my children | | | | |
| I am burdened by uncertainty about how to act correctly during the crisis | | | | |
| I am burdened by uncertainty about contact persons during the crisis | | | | |
| I am burdened by uncertainty about my financial situation during the crisis | | | | |
| I am burdened by uncertainty about the temporal scope of the crisis | | | | |
| I am burdened by a feeling of not being able to let patients down during the crisis | | | | |

**Over the last 2 weeks, how often have you been bothered by the following problems?**

| | Not at all | Several days | More than half the days | Nearly every day |
|---|---|---|---|---|
| Feeling nervous, anxious or on edge | | | | |
| Not being able to stop or control worrying | | | | |
| Little interest or pleasure in doing things | | | | |
| Feeling down, depressed or hopeless | | | | |
