# Supplementary material for: Pandemic-related attitudes, stressors and work outcomes among medical assistants during the SARS-CoV-2 (“Coronavirus”) pandemic in Germany: A cross-sectional Study
Source: PLoS One. 2021 Jan 14;16(1):e0245473. doi: 10.1371/journal.pone.0245473 (PMC7808691; doi:10.1371/journal.pone.0245473)
Supplement: S2 File — (PDF) [file pone.0245473.s002.pdf]

Fragebogen zur SARS-CoV-2 („Coronavirus“) Pandemie

1. **Geschlecht**
   - Männlich
   - Weiblich
   - Divers

2. **Geburtsjahr**
   - __________

3. **Haben Sie einen festen Partner/eine feste Partnerin?**
   - Ja
   - Nein

4. **Leben in Ihrem Haushalt betreuungspflichtige Personen?**
   - Ja, betreuungspflichtige Kinder
   - Ja, betreuungspflichtige Erwachsene
   - Nein

5. **Was ist Ihr höchster Schulabschluss?**
   - Haupt- oder Volksschulabschluss
   - Realschulabschluss / Mittlere Reife / Fachschulreife
   - Fachhochschulreife oder Abitur
   - Anderer Schulabschluss (z.B. im Ausland erworben)

6. **Arbeiten Sie momentan als (Zahn-) Medizinische/r Fachangestellte/r?**
   - Ja, als Medizinische/r Fachangestellte/r
   - Ja, als Zahnmedizinische/r Fachangestellte/r
   - Nein, ich arbeite als: ______________________
   - Ich bin momentan nicht erwerbstätig (z.B. arbeitssuchend, Mutterschutz)

7. **Wo arbeiten Sie momentan?**
   - Medizinisches Versorgungszentrum
   - Krankenhaus/Klinik
   - Reha-Bereich
   - Hausarztpraxis
   - Zahnarztpraxis
   - Facharztpraxis mit folgender Fachrichtung: ______________________________
   - Sonstiges, nämlich: ________________________

8. **Wie würden Sie Ihren Gesundheitszustand im Allgemeinen beschrieben?**
   - Sehr gut
   - Gut
   - Mittelmäßig
   - Schlecht
   - Sehr schlecht

9. **In meinem Familien-/Freundeskreis gibt es bestätigte oder vermutete Fälle von SARS-CoV-2.**
   - Ja
   - Nein

10. **Unter meinen Arbeitskollegen/*innen gibt es bestätigte oder vermutete Fälle von SARS-CoV-2.**
    - Ja
    - Nein

11. **Sie selbst sind bereits positiv auf SARS-CoV-2 getestet worden.**
    - Ja
    - Nein

Die folgenden Fragen beziehen sich auf die aktuelle SARS-CoV-2 Pandemie („Coronavirus"). Bitte geben Sie an, in wieweit Sie den folgenden Aussagen zustimmen.

	Stimme gar nicht zu	Stimme nicht zu	Stimme zu	Stimme voll zu
Ich fühle mich für den Umgang mit SARS-CoV-2 Patienten ausreichend durch den Arbeitgeber informiert.				
Ich fühle mich für den Umgang mit SARS-CoV-2 Patienten durch den Arbeitgeber ausreichend vorbereitet.				
Die SARS-CoV-2 Pandemie wird von meinem Arbeitgeber ernst genommen.				
Die Wahrscheinlichkeit einer Infektion mit SARS-CoV-2 ist für mich höher als für eine Person gleichen Alters und Geschlechts aus der Allgemeinbevölkerung.				
Mein Arbeitsaufkommen ist bedingt durch die SARS-CoV-2 Pandemie gestiegen.				
Bedingt durch die SARS-CoV-2 Pandemie leidet die Versorgung von Patienten mit anderweitigen Erkrankungen.				
Auf meiner Arbeit sind alle nötigen Materialien zum persönlichen Schutz vor SARS-CoV-2 ausreichend für mich verfügbar.				
Ich kann diese Materialien so nutzen, dass ich mich ausreichend geschützt vor einer Infektion mit SARS-CoV-2 fühle.				
Mich belasten Gedanken um eine mögliche Infektion mit SARS-CoV-2 während der Arbeitszeit.				
Mich belastet der krisenbedingte Ausfall von Kollegen/Mitarbeitern auf der Arbeit.				
Mich belastet die Betreuungssituation meiner Kinder.				
Mich belastet Ungewissheit über korrektes Handeln in der Krise.				
Mich belastet Ungewissheit über Ansprechpartner in der Krise.				
Mich belastet Ungewissheit über meine finanzielle Situation in der Krise.				
Mich belastet Ungewissheit über das zeitliche Ausmaß der Krise.				
Mich belastet das Pflichtgefühl gegenüber meinen Patienten („nicht im Stich lassen dürfen") in der Krise.				

Wie oft fühlten Sie sich im Verlauf der letzten 2 Wochen durch die folgenden Beschwerden beeinträchtigt?

	**Überhaupt nicht**	**An einzelnen Tagen**	**An mehr als der Hälfte der Tage**	**Beinahe jeden Tag**
Nervosität, Ängstlichkeit oder Anspannung				
Nicht in der Lage sein, Sorgen zu stoppen oder zu kontrollieren				
Wenig Interesse oder Freude an Ihren Tätigkeiten				
Niedergeschlagenheit, Schwermut oder Hoffnungslosigkeit				
