# Supplementary material for: Pandemic-related attitudes, stressors and work outcomes among medical assistants during the SARS-CoV-2 (“Coronavirus”) pandemic in Germany: A cross-sectional Study
Source: PLoS One. 2021 Jan 14;16(1):e0245473. doi: 10.1371/journal.pone.0245473 (PMC7808691; doi:10.1371/journal.pone.0245473)
Supplement: S1 Table — (DOCX) [file pone.0245473.s003.docx]

**S1 Table. Sensitivity Analysis: Logistic regression results for SARS-CoV-2 related attitudes among medical assistants (n= 2,150) without adjusting for depression and anxiety disorder**

|  | SARS-CoV-2 related attitudes | | | | | | | | |
| --- | --- | --- | --- | --- | --- | --- | --- | --- | --- |
|  | Higher perceived risk of contraction |  | Feeling of sufficient protection from infection |  | Feeling sufficiently prepared |  | Feeling sufficiently informed |  | Increased workload due to pandemic |
|  | OR  (95% CI) |  | OR  (95% CI) |  | OR  (95% CI) |  | OR  (95% CI) |  | OR  (95% CI) |
| Age |  |  |  |  |  |  |  |  |  |
| 33-42 (vs. 18-32) | 1.13 (0.85-1.52) |  | 1.04 (0.82-1.33) |  | 1.00 (0.79-1.26) |  | 0.99 (0.77-1.26) |  | 1.14 (0.91-1.45) |
| 43 and older (vs. 18-32) | 1.34 (1.01-1.77) |  | 0.93 (0.74-1.16) |  | 0.98 (0.79-1.22) |  | 0.92 (0.74-1.15) |  | 1.00 (0.80-1.24) |
| Permanent Partner |  |  |  |  |  |  |  |  |  |
| Yes (vs. no) | 0.93 (0.68-1.28) |  | 0.87 (0.67-1.11) |  | 0.89 (0.70-1.14) |  | 0.86 (0.66-1.11) |  | 0.73 (0.57-0.93) |
| Children under care in same household |  |  |  |  |  |  |  |  |  |
| Yes (vs. no) | 0.99 (0.78-1.28) |  | 0.77 (0.62-0.95) |  | 0.98 (0.81-1.20) |  | 1.06 (0.86-1.30) |  | 1.02 (0.84-1.25) |
| Highest level of education |  |  |  |  |  |  |  |  |  |
| Intermediate^2^ (vs. low^1^) | 0.88 (0.55-1.40) |  | 0.70 (0.50-1.00) |  | 0.84 (0.59-1.20) |  | 0.93 (0.64-1.34) |  | 0.88 (0.62-1.24) |
| High^3^ (vs. low^1^) | 0.88 (0.53-1.48) |  | 0.67 (0.45-0.99) |  | 0.76 (0.51-1.13) |  | 0.80 (0.53-1.20) |  | 0.69 (0.47-1.03) |
| Place of work |  |  |  |  |  |  |  |  |  |
| Specialist practice (vs. general   practice) | 0.75 (0.59-0.95) |  | 0.86 (0.70-1.04) |  | 0.65 (0.54-0.79) |  | 0.55 (0.45-0.67) |  | 0.37 (0.31-0.45) |
| Medical care center (vs. general   practice) | 1.24 (0.73-2.10) |  | 0.96 (0.65-1.43) |  | 0.90 (0.62-1.33) |  | 0.48 (0.33-0.70) |  | 0.38 (0.26-0.55) |
| Hospital/clinic (vs. general practice) | 1.19 (0.61-2.35) |  | 1.37 (0.84-2.23) |  | 0.73 (0.45-1.19) |  | 1.00 (0.59-1.71) |  | 0.44 (0.27-0.72) |
| Other (vs. general practice) | 1.33 (0.65-2.73) |  | 0.93 (0.55-1.56) |  | 0.64 (0.40-1.05) |  | 0.56 (0.34-0.91) |  | 0.58 (0.36-0.93) |
| Self-rated health |  |  |  |  |  |  |  |  |  |
| Good (vs. bad) | 0.60 (0.44-0.82) |  | 1.94 (1.51-2.49) |  | 1.85 (1.49-2.29) |  | 1.81 (1.45-2.26) |  | 0.73 (0.58-0.91) |
| SARS-CoV-2 cases among friends and family |  |  |  |  |  |  |  |  |  |
| Yes (vs. no) | 1.31 (0.94-1.83) |  | 1.02 (0.80-1.32) |  | 0.91 (0.72-1.16) |  | 0.81 (0.63-1.04) |  | 1.14 (0.89-1.45) |
| SARS-CoV-2 cases among colleagues |  |  |  |  |  |  |  |  |  |
| Yes (vs. no) | 1.14 (0.81-1.60) |  | 1.01 (0.78-1.32) |  | 0.69 (0.54-0.88) |  | 0.80 (0.62-1.04) |  | 1.65 (1.27-2.13) |
| Own previous infection with SARS-CoV-2 |  |  |  |  |  |  |  |  |  |
| Yes (vs. no) | 0.84 (0.28-2.55) |  | 0.57 (0.21-1.58) |  | 0.81 (0.34-1.93) |  | 0.89 (0.36-2.18) |  | 3.54 (1.15-10.86) |

OR Odds ratio; CI Confidence interval; 1: Low: secondary modern school qualification (‘Haupt-/Volksschulabschluss’); 2: Intermediate: secondary school level I certificate (‘Mittlere Reife’, ‘Realschulabschluss’ or ‘Fachschulreife’); 3: High: general qualification for university entrance (‘Abitur’) or entrance qualification limited to universities of applied sciences (‘Fachhochschulreife’)
