# Supplementary material for: Pandemic-related attitudes, stressors and work outcomes among medical assistants during the SARS-CoV-2 (“Coronavirus”) pandemic in Germany: A cross-sectional Study
Source: PLoS One. 2021 Jan 14;16(1):e0245473. doi: 10.1371/journal.pone.0245473 (PMC7808691; doi:10.1371/journal.pone.0245473)
Supplement: S2 Table — (DOCX) [file pone.0245473.s004.docx]

**S2 Table.** **Sensitivity Analysis: Logistic regression results for SARS-CoV-2 related stressors among medical assistants (n= 2,150) without adjusting for depression and anxiety disorder**

|  | SARS-CoV-2 related stressors | | | | | | |
| --- | --- | --- | --- | --- | --- | --- | --- |
|  | Thoughts about contraction at workplace |  | Shortfall of colleagues |  | Childcare situation* |  | Not being able to let patients down |
|  | OR  (95% CI) |  | OR  (95% CI) |  | OR  (95% CI) |  | OR  (95% CI) |
| Age |  |  |  |  |  |  |  |
| 33-42 (vs. 18-32) | 0.72 (0.57-0.92) |  | 0.97 (0.77-1.22) |  | 1.13 (0.67-1.88) |  | 0.72 (0.55-0.94) |
| 43 and older (vs. 18-32) | 0.88 (0.70-1.10) |  | 0.99 (0.80-1.23) |  | 0.24 (0.14-0.40) |  | 0.82 (0.64-1.05) |
| Permanent Partner |  |  |  |  |  |  |  |
| Yes (vs. no) | 1.21 (0.94-1.55) |  | 1.15 (0.91-1.47) |  | 1.80 (1.02-3.17) |  | 0.95 (0.72-1.25) |
| Children under care in same household |  |  |  |  |  |  |  |
| Yes (vs. no) | 1.30 (1.05-1.60) |  | 0.91 (0.75-1.11) |  | - |  | 1.15 (0.92-1.44) |
| Highest level of education |  |  |  |  |  |  |  |
| Intermediate^2^ (vs. low^1^) | 0.74 (0.50-1.08) |  | 0.85 (0.60-1.20) |  | 0.88 (0.43-1.80) |  | 0.69 (0.44-1.08) |
| High^3^ (vs. low^1^) | 0.62 (0.40-0.94) |  | 0.87 (0.59-1.28) |  | 1.04 (0.47-2.29) |  | 0.49 (0.30-0.79) |
| Place of work |  |  |  |  |  |  |  |
| Specialist practice (vs. general   practice) | 1.29 (1.06-1.57) |  | 1.16 (0.96-1.40) |  | 1.41 (0.97-2.03) |  | 0.78 (0.63-0.97) |
| Medical care center (vs. general   practice) | 0.94 (0.63-1.38) |  | 1.42 (0.97-2.08) |  | 0.81 (0.38-1.74) |  | 0.89 (0.57-1.38) |
| Hospital/clinic (vs. general practice) | 1.14 (0.68-1.89) |  | 1.53 (0.91-2.56) |  | 1.50 (0.50-4.48) |  | 0.62 (0.36-1.05) |
| Other (vs. general practice) | 1.38 (0.81-2.35) |  | 1.20 (0.73-1.97) |  | 1.66 (0.61-4.52) |  | 0.83 (0.48-1.46) |
| Self-rated health |  |  |  |  |  |  |  |
| Good (vs. bad) | 0.36 (0.27-0.47) |  | 0.58 (0.46-0.73) |  | 0.77 (0.49-1.20) |  | 0.56 (0.42-0.74) |
| SARS-CoV-2 cases among friends and family |  |  |  |  |  |  |  |
| Yes (vs. no) | 1.23 (0.95-1.59) |  | 0.85 (0.67-1.09) |  | 0.80 (0.51-1.25) |  | 1.13 (0.85-1.51) |
| SARS-CoV-2 cases among colleagues |  |  |  |  |  |  |  |
| Yes (vs. no) | 1.28 (0.98-1.69) |  | 2.63 (2.01-3.45) |  | 1.77 (1.00-3.16) |  | 1.32 (0.97-1.79) |
| Own previous infection with SARS-CoV-2 |  |  |  |  |  |  |  |
| Yes (vs. no) | 1.16 (0.46-2.95) |  | 1.77 (0.70-4.49) |  | 0.19 (0.04-0.88) |  | 1.04 (0.38-2.90) |

OR Odds ratio; CI Confidence interval; 1: Low: secondary modern school qualification (‘Haupt-/Volksschulabschluss’); 2: Intermediate: secondary school level I certificate (‘Mittlere Reife’, ‘Realschulabschluss’ or ‘Fachschulreife’); 3: High: general qualification for university entrance (‘Abitur’) or entrance qualification limited to universities of applied sciences (‘Fachhochschulreife’)

**Supplementary Table 2 (continued). Sensitivity Analysis: Logistic regression results for SARS-CoV-2 related stressors among medical assistants (n= 2,150) without adjusting for depression and anxiety disorder**

|  | SARS-CoV-2 related stressors | | | | | | |
| --- | --- | --- | --- | --- | --- | --- | --- |
|  | Uncertainty about acting correctly |  | Uncertainty about contact persons |  | Uncertainty about financial situation |  | Uncertainty about temporal scope |
|  | OR  (95% CI) |  | OR  (95% CI) |  | OR  (95% CI) |  | OR  (95% CI) |
| Age |  |  |  |  |  |  |  |
| 33-42 (vs. 18-32) | 0.78 (0.59-1.03) |  | 1.08 (0.85-1.39) |  | 1.02 (0.80-1.31) |  | 0.61 (0.35-1.06) |
| 43 and older (vs. 18-32) | 0.64 (0.50-0.83) |  | 0.95 (0.76-1.19) |  | 0.88 (0.70-1.10) |  | 0.51 (0.32-0.83) |
| Permanent Partner |  |  |  |  |  |  |  |
| Yes (vs. no) | 1.17 (0.89-1.55) |  | 1.09 (0.85-1.41) |  | 0.99 (0.77-1.27) |  | 1.30 (0.79-2.13) |
| Children under care in same household |  |  |  |  |  |  |  |
| Yes (vs. no) | 1.34 (1.06-1.70) |  | 1.14 (0.92-1.40) |  | 1.49 (1.21-1.84) |  | 2.43 (1.47-4.02) |
| Highest level of education |  |  |  |  |  |  |  |
| Intermediate^2^ (vs. low^1^) | 0.47 (0.28-0.78) |  | 0.76 (0.52-1.12) |  | 0.75 (0.52-1.11) |  | 1.32 (0.66-2.64) |
| High^3^ (vs. low^1^) | 0.35 (0.20-0.61) |  | 0.56 (0.37-0.86) |  | 0.71 (0.47-1.09) |  | 1.44 (0.64-3.24) |
| Place of work |  |  |  |  |  |  |  |
| Specialist practice (vs. general   practice) | 1.04 (0.83-1.31) |  | 1.06 (0.87-1.29) |  | 1.31 (1.08-1.60) |  | 0.80 (0.52-1.23) |
| Medical care center (vs. general   practice) | 0.86 (0.55-1.34) |  | 0.69 (0.47-1.01) |  | 0.94 (0.64-1.38) |  | 0.77 (0.33-1.75) |
| Hospital/clinic (vs. general practice) | 0.93 (0.53-1.65) |  | 0.99 (0.56-1.64) |  | 0.73 (0.45-1.19) |  | 1.23 (0.36-4.19) |
| Other (vs. general practice) | 0.78 (0.45-1.36) |  | 0.88 (0.53-1.46) |  | 1.13 (0.67-1.89) |  | 0.81 (0.28-2.34) |
| Self-rated health |  |  |  |  |  |  |  |
| Good (vs. bad) | 0.43 (0.32-0.59) |  | 0.47 (0.36-0.60) |  | 0.59 (0.46-0.76) |  | 0.52 (0.29-0.94) |
| SARS-CoV-2 cases among friends and family |  |  |  |  |  |  |  |
| Yes (vs. no) | 1.10 (0.82-1.48) |  | 1.06 (0.82-1.37) |  | 1.00 (0.77-1.28) |  | 0.85 (0.50-1.45) |
| SARS-CoV-2 cases among colleagues |  |  |  |  |  |  |  |
| Yes (vs. no) | 1.29 (0.94-1.77) |  | 1.23 (0.94-1.62) |  | 0.98 (0.75-1.27) |  | 0.86 (0.50-1.50) |
| Own previous infection with SARS-CoV-2 |  |  |  |  |  |  |  |
| Yes (vs. no) | 1.40 (0.46-4.24) |  | 2.17 (0.72-6.54) |  | 0.92 (0.38-2.25) |  | 0.66 (0.15-3.01) |

OR Odds ratio; CI Confidence interval; 1: Low: secondary modern school qualification (‘Haupt-/Volksschulabschluss’); 2: Intermediate: secondary school level I certificate (‘Mittlere Reife’, ‘Realschulabschluss’ or ‘Fachschulreife’); 3: High: general qualification for university entrance (‘Abitur’) or entrance qualification limited to universities of applied sciences (‘Fachhochschulreife’)
